# The impact of restricted grazing systems on the behaviour and welfare of ponies

**DOI:** 10.1111/evj.14411

**Published:** 2024-09-14

**Authors:** Roxane Kirton, Imogen Sandford, Eleanor Raffan, Sarah Hallsworth, Oliver H. P. Burman, Ruth Morgan

**Affiliations:** ^1^ Redwings Horse Sanctuary Norwich UK; ^2^ School of Life & Environmental Sciences, Joseph Banks Laboratories University of Lincoln Lincoln UK; ^3^ Department of Physiology, Development, and Neuroscience University of Cambridge Cambridge UK; ^4^ Scotland's Rural College Edinburgh UK; ^5^ Royal (Dick) School of Veterinary Studies University of Edinburgh Roslin UK

**Keywords:** grazing, horse, obesity, weight loss, welfare

## Abstract

**Background:**

Equine obesity is a growing concern. Much of the current management advice centres on dietary restrictions, including the removal or limitation of grazing. Little is known about the impact of these approaches on the welfare of the horse.

**Objective:**

This study investigates the effect of two commonly used grazing systems advocated for the control of weight—the ‘strip‐grazing’ and the ‘track’ systems—on the behaviour and welfare of outdoor‐living ponies.

**Study design:**

A within‐subject cross‐over experimental design with four groups of pasture‐kept ponies experiencing each system for 4 weeks in a random order.

**Methods:**

Time budgets and behavioural indicators of welfare were measured using 24‐h electronic surveillance, morphometric parameters including weight, body condition score and cresty neck score were measured weekly and activity levels were tracked. The effect of grazing system on movement and behaviour was tested using a general linear model.

**Results:**

Ponies moved more [median (IQR) % time spent moving, track: 3.23% (2.08%), strip: 2.02% (0.90%); *p* = 0.001] and travelled a greater distance [median (IQR) metres/24 h, track: 7013.47 m (1761.49 m), strip: 5331.91 m (494.16 m); *p* < 0.001] and engaged in less overt agonistic behaviour on the track system compared with the strip system [median (IQR) prevalence per hour; track: 0.14 (0.30), strip: 0.21 (0.37) *p* = 0.02].

**Main limitations:**

A relatively short time period of exposure to each grazing system.

**Conclusions:**

Ponies on strip systems moved less and exhibited increased agonistic interactions compared with the track system, maybe as a result of a perceived reduction in space or concentration of resources, although the accessible areas were matched. These results suggest that there may be physical as well as psychological health benefits to the track system.

## 1 INTRODUCTION

Obesity is an increasing welfare concern in leisure horses, with 31.2% of horses identified as being obese in one owner‐reported survey in Britain.^1^ Equine obesity is associated with significant health concerns, including equine metabolic syndrome (EMS) and laminitis.^2^ Risk factors for developing obesity include the role/use of the horse, with more leisure horses being obese, and breed with an increased prevalence in British native breeds.^1^, ^3^, ^4^, ^5^, ^6^

The mainstay for the treatment of EMS and other obesity‐related conditions is primarily focused on weight loss,^7^ which, if sufficient, can result in a complete reversal of the associated metabolic dysfunction.^2^ Recommendations for weight loss centre around restricting dietary intake, which often involves a reduction in forage consumption and restriction of grazing.^8^, ^9^, ^10^, ^11^ Grazing is a central and innate facet of equine behaviour, and horses graze for approximately two thirds of their 24 h time budgets if allowed^12^ and, in the wild, demonstrate clear diurnal rhythms to grazing.^12^ The nature, size, quantity and timing of the grazing available, therefore, has an impact not only on grazing behaviour but consequently on all other behaviours.^13^, ^14^ Grazing and movement are innate evolved behaviours which have allowed the horse to occupy its ecological niche and could be described as ethological needs.^15^ If either is restricted, the impact on the behavioural needs may lead to frustration and expression of rebound behaviours which may be problematic and/or abnormal,^15^ such as increased aggression and stereotypic behaviour or increased food intake.^6^, ^16^ The duration of the restrictions required to achieve the desired weight loss goal is often significant, compounding any compromises to welfare further. In addition, a decrease in efficacy of dietary restriction over time has been shown in both humans and horses,^5^, ^17^ requiring more extreme levels of restriction if this method is to remain effective. Owners can also struggle with compliance with weight control programmes for their horses, which may be due to several factors, including practical limitations, adverse responses and unwanted horse behaviour.^10^

In the United Kingdom, the ‘strip’ system is the most common form of restricted grazing which owners implement.^18^ The strip system involves limiting the grazing area to a, usually rectangular, portion of the field and increasing the size of this area over time by moving the fencing. Recent work has shown that digestible energy intake is reduced when ponies initially go onto strip grazing compared with free grazing^19^ and that strip grazing can potentially limit weight gain in ponies.^20^ A great number of owners also expressed a desire to try the ‘track’ system^18^ and this system is the most common ‘alternative’ grazing system used.^21^ The ‘track’ system involves creating a track around the perimeter of the grazing area, which can be increased in size gradually and it is designed to encourage movement, though current data available is not conclusive on this.^22^ Data relating to track systems remains very limited^23^ and there is little information comparing the behavioural impact of these systems.

The aim of this study was to determine the impact strip and track grazing systems have on the behaviour and grazing patterns of outdoor‐living ponies. It was hypothesised that the track system would allow for move free movement of the animals, would have fewer negative impacts on behaviour and would disrupt the natural rhythm of grazing less.

## 2 MATERIALS AND METHODS

### 2.1 Animals

A convenience sample of 35 outdoor‐living ponies kept in four herds in the east of England was included in the study. The herds were on pasture 24 h a day without any supplemental feeding and had lived together for a minimum of 3 months before the onset of the study so that group social relationships were considered to be stable and well established. Routine preventative health care, veterinary requirements and pasture management were delivered as normal during the period of the study. The size of the groups ranged from 7 to 11 ponies (Group 1 *n* = 7, Group 2 *n* = 11, Group 3 *n* = 10 and Group 4 *n* = 7) and groups were either geldings only (Groups 1 and 4) or a mixture of mares and geldings (Groups 2 and 3). 27% of the ponies were mares and 71% were geldings. All ponies included were either native types or cob types (<14.3hh) with a mean age of 6.8 years, ranging from 4 to 13 years.

### 2.2 Study design

The study was conducted during July and August 2019 when grazing was sufficient to provide all dietary requirements. A within‐subject study design was used with each group experiencing 4 weeks living on a strip system and 4 weeks living on a track system in a randomly allocated order determined using an online randomising tool (randomer.org).

**FIGURE 1:**
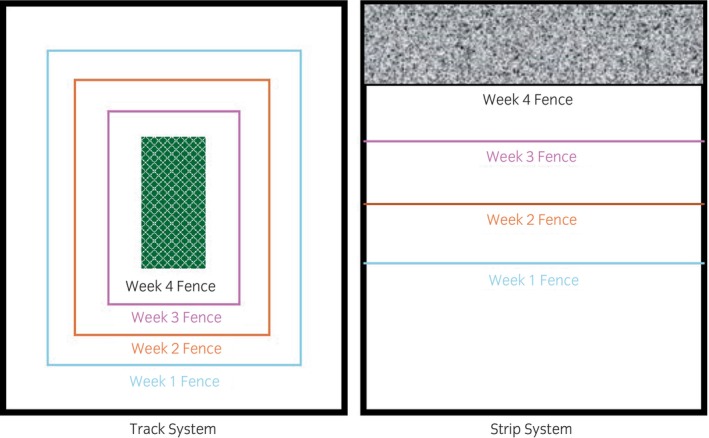
A diagrammatic representation of the weekly space allocation on the track system and strip systems. Both the initial grazing area and each weekly addition are area‐matched across both systems for each group. The white hatched areas indicate areas of unused grazing for each system.

The grazing areas accessible to the ponies initially were calculated in hectares using field management software (Gatekeeper, Farmplan) and matched so they were the same on both grazing systems for each group (Figure 1). GPS coordinates were used to position the electric fencing that was erected to create the different systems within the allocated fields. All ponies were habituated to electric fencing before the study's onset. Eight fields were used, and all fields were rested for over a month before use to ensure adequate and consistent grass coverage. The electric fencing was moved once weekly on the track system and twice weekly on the strip system when required.

### 2.3 Morphometric measures

#### 2.3.1 Bodyweight

Bodyweight was measured in kilogrammes using a mobile weighbridge brought to the field (Tokyo Thoroughbred, Horse Weigh®) at the start, end and weekly for the duration of the study. Groups were weighed in a randomly selected order each week (randomer.org), individuals were weighed in the order in which they presented to handlers.

#### 2.3.2 Body condition score and cresty neck score

Body condition score (BCS) and cresty neck score (CNS) were recorded at the same time as weight. BCS was scored on a 0–5 scale^24^ and CNS on a 0–5 scale^25^ by a single trained and experienced operator.

#### 2.3.3 Behavioural observations

A mobile video surveillance system was used to record the behaviour of each group for a 24‐h period once weekly on a randomly allocated day, avoiding Mondays due to the collection of morphometric measurements. Four 4‐megapixel motorised varifocal lens bullet cameras (Hikvision, Farmwatch Ltd.) were attached to 3 m poles and secured to fence posts around the field perimeter positioned to achieve the best coverage of the entire grazing area. A 4‐channel network video recorder 1 TB hard drive (Hikvision, Farmwatch Ltd.) was used for recording and playback of all video footage. Infrared floodlights were used during darkness to increase the night vision scope of the cameras. Instantaneous scan sampling^26^ of all visible ponies was performed at 15‐min intervals throughout the 24 h recording period and behaviour was categorised according to the following ethogram: grazing, browsing, walking, trotting, cantering, galloping, drinking, standing, sternal recumbency, lateral recumbency, self‐grooming, urination, defecation, play, allogrooming, overt agonistic interactions and stereotypic behaviour (Table S1). We also used continuous observation to record all instances of the behaviours thought to be most indicative of welfare status, both positive (play and allogrooming) and negative (overt agonistic interactions and stereotypic behaviour).^27^, ^28^, ^29^

Time budgets of behaviours (total number of recordings of each of the behaviours divided by the total number of recordings of ponies visible) were calculated as percentages. The hourly prevalence rate of behaviours indicative of welfare status was calculated.

Galloping was never recorded so was removed from the analysis. Trotting and cantering also occurred infrequently so were combined with walking to form an ‘ambulating’ behavioural category. Defecation and urination were rarely recorded and so were combined into a single ‘elimination’ category.

#### 2.3.4 Activity levels

Distance travelled per 24 h was recorded in metres using GPS dataloggers (tsi Transystem GL‐770) attached to headcollars. Four ponies from each group were pseudo‐randomly selected after excluding those individuals considered to be unsuitable, either due to health reasons or because they were not habituated to wearing a headcollar. A GPS unit was used to record movement for each pony over a 24‐h period once a week on a randomly allocated day, excluding Mondays (see above). Each GPS unit was attached for a minimum of 24.5 h so that the first and last 10 min of recording could be discarded because it incorporated the fitting and removal of the headcollar. Recordings of less than 1‐h were removed from the data set. A weekly group average was calculated from all remaining data and used for statistical analysis.

#### 2.3.5 Data analysis

All statistical analysis was performed using Minitab version 17. The normality of all data was assessed using Anderson–Darling tests and any data found to be non‐normally distributed were log transformed. Where data were not normally distributed, all descriptive statistics and graphical representations are presented in medians and interquartile ranges. BCS and CNS values were analysed as ordinal data.

The effects of the grazing system (Strip, Track), week (1–4) and their interaction were evaluated for each of the dependent variables (time budget and activity). Initial analysis of each data set was performed using a general linear model (GLM) with the grazing system, week and their interaction as fixed factors and the group included as a random factor. Where week was found to significantly influence the dependent variable, further post hoc evaluation using a pairwise comparison Tukey test was used to evaluate any differences between weeks. Significant interactions between a week and the grazing system were examined for each week separately to reveal whether there was a difference between the two grazing systems, using a GLM with the grazing system as a factor. In addition, both grazing systems were examined separately to see if there was an overall difference between the 4 weeks, using a GLM with week as a factor. Where significant results were found, differences between individual weeks were further examined using a pairwise comparison Tukey test. Statistical significance was taken as *p* < 0.05.

Grazing behaviour was further investigated for the presence of a circadian rhythm. Each 24‐h time sampling period was split into four time periods: morning, afternoon, evening and night. The morning was defined as the period between sunrise and midday. Afternoon was defined as the period between midday and 5:00 pm. Evening was defined as the period between 5:00 pm and sunset. Night was defined as the period between sunset and sunrise. The daily sunrise and sunset times were acquired from the Met Office (Weather and climate change—Met Office). Percentage time spent grazing was determined by taking the total grazing behaviour in a time period and dividing by the total grazing behaviour observed in the complete 24‐h period. To establish whether the percentage of the total time spent grazing was significantly different between time periods and then between grazing systems across a 24‐h period, a two‐way repeated ANOVA with appropriate post hoc tests was performed.

## 3 RESULTS

### 3.1 Bodyweight and condition scores (BCS, CNS)

Across all groups, the median BCS at the start of the study was 4.5 [interquartile range (IQR) 2] and the prevalence of obesity (BCS ≥4/5) was 83%. Median CNS at the start of the study was 3 (IQR 2). We found no significant differences between ‘weeks’, ‘grazing system’ or their interaction for any of the morphometric measurement changes: bodyweight (grazing system; *p* = 0.8, week; *p* = 0.6, interaction between week and grazing system; *p* = 0.4), BCS (grazing system; *p* = 0.5, week; *F*_3,21_ = 0.76, *p* = 0.5, interaction between week and grazing system; *p* = 0.4) and CNS change (grazing system; *p* = 0.8, week; *p* = 0.3, interaction between week and grazing system; *p* = 0.5).

### 3.2 Behavioural observations

In both systems, ponies spent the majority of their time grazing (Table 1). Ponies spent more time ambulating on the track system than on the strip system [median percentage of 24 h (IQR), track: 3.23% (2.08%), strip: 2.02% (0.90%, *p* = 0.001)]. There was a significant effect of week on ambulatory activity on the track system but not the strip, with an increase in ambulation over time peaking at Week 3 (*F*_3,11_ = 6.58, *p* = 0.008) and variations seen between Weeks 1 and 3 [median (IQR), Week 1: 1.82% (1.65%) and Week 3: 5.83% (2.84%), *p* = 0.02] and Weeks 2 and 3 [median (IQR), Week 2: 2.23% (1.27%) and Week 3: 5.83% (2.84%), *p* = 0.01].

**TABLE 1:** Overall time budgets (%) for each grazing system with normally distributed data displayed as mean (±StDev) and non‐normally data displayed as median (IQR).

System	Grazing	Browsing	Ambulating	Standing	Sternal	Lateral	Self‐grooming	Drinking	Elimination (urinate and defaecation)
Strip	65.28 (±4.57)	0.40 (IQR 1.19)	2.02* (IQR 0.90)	21.97 (±4.75)	4.51 (IQR 1.74)	1.01 (IQR 1.6)	0.54 (±0.38)	0.44 (±0.34)	0.13 (IQR 0.16)
Track	64.73 (±6.94)	0.67 (IQR 2.71)	3.23* (IQR 2.08)	19.05 (±6.57)	3.95 (IQR 1.91)	0.68 (IQR 1.43)	0.37 (±0.30)	0.51 (±0.40)	0.12 (IQR 0.17)

*Note*: Statistically significant results when the grazing systems were compared are indicated with an asterisk.

### 3.3 Key behavioural indicators of welfare

Overt agonistic behaviour was increased in the strip grazing system compared with the track system [median per hour (IQR); track 0.14 (0.30) v strip 0.21 (0.37, *p* = 0.02)], no other behaviours differed (Table 2). Week had a significant effect on the prevalence of overt agonistic behaviour (*F*_3,22_ = 15.96, *p* < 0.001) with the highest rates seen in Week 1 and reductions in subsequent weeks [median (IQR), Week 1: 0.50 per hour (0.12 per hour), Week 2: 0.21 (0.39 per hour), Week 3: 0.14 per hour (0.12 per hour) and Week 4: 0.11 per hour (0.16 per hour)].

**TABLE 2:** Prevalence rates of welfare indicator behaviours (per hour) for each grazing system with normally distributed data displayed as mean (±StDev) and non‐normally data displayed as median (IQR).

System	Play	Stereotypic	Overt agonistic	Allogrooming
Strip	0.13 (IQR 0.17)	0.01 (±0.03)	0.21 (IQR 0.37)*	0.68 (±0.54)
Track	0.13 (IQR 0.12)	0	0.14 (IQR 0.30)*	0.53 (±0.39)

*Note*: Due to the very low prevalence rate of stereotypic behaviour this is also displayed as mean (StDev). Statistically significant results when the grazing systems were compared are indicated with an asterisk.

The week was found to impact the prevalence of allogrooming performed (*p* = 0.02) and, although there was no significant difference between any individual pairs of weeks, the highest rate occurred in Week 1 followed by a tendency to reduce in Weeks 2 and 3 [mean (±SD), Week 1: 0.97 per hour (±0.23 per hour), Week 2: 0.56 per hour (±0.13 per hour), Week 3: 0.47 per hour (±0.13 per hour), Week 4: 0.47 per hour (±0.15 per hour)].

### 3.4 Activity levels

**FIGURE 2:**
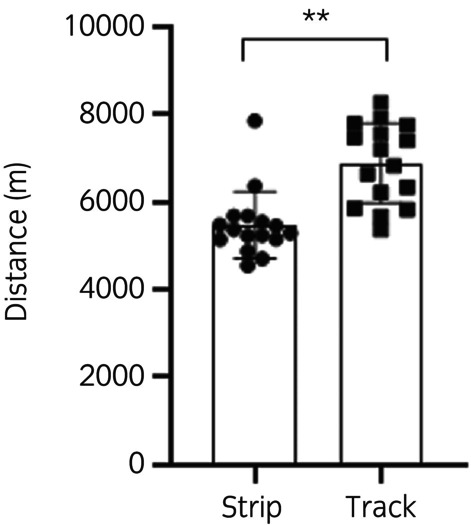
Distance travelled in metres when ponies were managed on a track system compared with an area‐matched strip system (*p < 0.001). Data are median (IQR).

Ponies moved a greater distance in 24 h on the track system than on the strip system (Figure 2) median (IQR), track system: 7013.47 m (1761.49 m), strip system: 5331.91 m (494.16 m, *p* < 0.001). There was no significant effect of the week (*p* = 0.2).

### 3.5 Grazing behaviour

**FIGURE 3:**
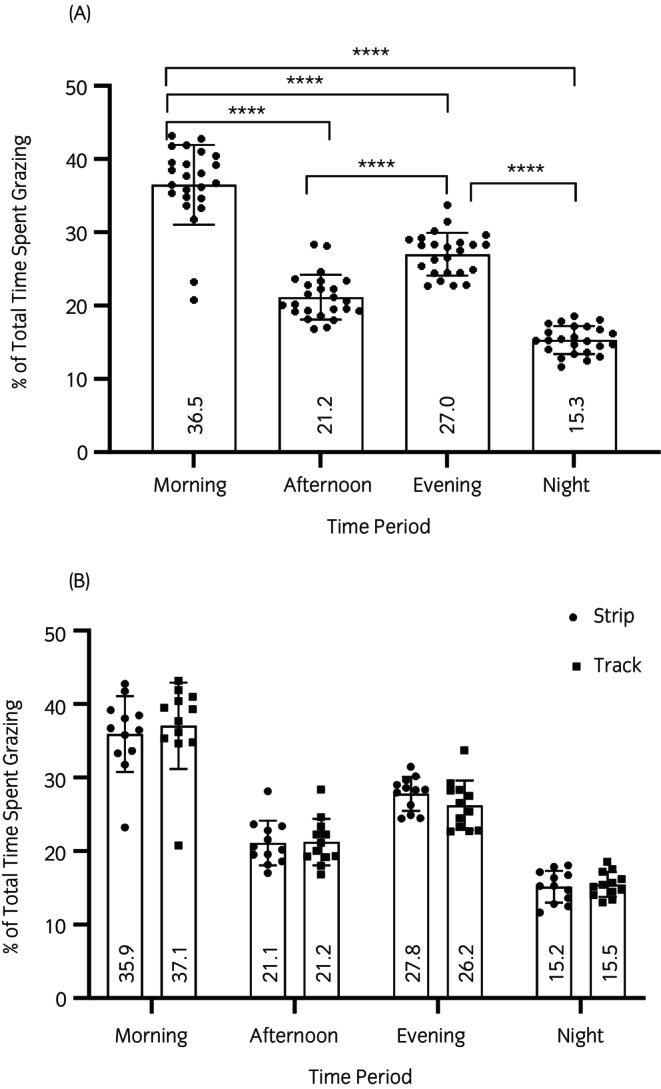
Percentage of total time spent grazing by all animals in the study combined (A) and animals divided into strip or track grazing systems (B). There was a difference in the time spent grazing during different periods of recording (****p < 0.001). Data are mean ± standard deviation.

The grazing behaviour demonstrated a distinct bimodal circadian rhythm with increased grazing in the morning and in the evening and less in the afternoon and at night (*p* < 0.001) (Figure 3A). This rhythm was maintained across the two grazing systems (Figure 3B) and there were no differences between the two (*p* > 0.9).

## 4 DISCUSSION

With obesity affecting almost 40% of leisure horses, management strategies require scrutiny to ensure they do not inadvertently introduce more problems for the horses being managed and the owners and veterinary professionals implementing the strategies. The mainstay of treatment for obesity is weight loss through reducing calorie intake and, if sufficient, this can successfully reverse many of the metabolic consequences.^2^ Reducing the intake of horses kept on grass can often be difficult but is preferable to long‐term stabling from a welfare standpoint, which concept owners appear to appreciate.^18^ Ideal management systems should provide for both physical and psychological good health. The most commonly used method of restrictive grazing implemented in the United Kingdom is strip grazing, but owners also report using the track systems and, interestingly, show a desire to try this system in the future because of the perceived improved welfare it offers.^18^ In this study, we considered the effect of two different grazing systems on behaviour and welfare. Our findings support the hypothesis that the track system appears to promote improved behaviour and welfare in line with owner perception.

Despite the use of these systems for weight reduction, we did not observe significant changes in the morphometric parameters (bodyweight, BCS and CNS) measured, which is perhaps unsurprising given the relatively short period of this study (4 weeks in each grazing system), and the levels of dietary restriction were unlikely to be as severe as those used in other weight management studies due to continuous access to grass. Weight loss studies have shown that even with significant dietary restrictions, effective weight loss can take several months.^10^ Increased risk of obesity in noncompetition animals may be associated with increased amounts of turnout time^1^, ^30^ indicating that weight is harder to manage on pasture. Ponies should have a natural variation in bodyweight according to the season, with increasing bodyweight in the summer grazing season and weight loss in winter when food is sparse and living conditions are tougher.^3^ It stands to reason that achieving weight loss in outdoor‐living ponies in summer is going to be challenging and, realistically, the aim of long‐term weight control should be to minimise weight gain in the summer and optimise weight loss in the winter.

Horses are herbivorous trickle feeders that spend a large proportion of their day grazing.^31^ Breaks between grazing bouts are relatively short and fasting does not typically last for more than 4 h.^32^ Horses naturally spend much of the day moving over vast distances between resources. The ponies in this study grazed for around 68% of their time on both systems in line with other studies.^33^ They also demonstrated a range of behaviours, including ambulating, predominantly at walking, browsing, self and allogrooming and lying down. The time budgets found in this study for both systems are consistent with those in previous studies looking at domestic horses in free and strip grazing.^12^, ^33^ There was, however, a significant increase in the daily ambulation and distance travelled on the area‐matched track system compared with the strip system in our study, suggesting increased voluntary exercise on the track system. Previous studies vary in their findings regarding voluntary exercise but Hampson et al. reported that larger paddock sizes were correlated with increased distances moved. However, free‐roaming horses moved on average 17.9 km/day, which is still substantially more than the 7.2 km/day achieved by those on large 16 ha paddocks.^22^ Free moving horses in the Australian outback can travel up to 28.3 km in a day.^34^ Increasing movement is likely to increase calorie expenditure which can only be of benefit to obese animals.

The effect of internal fence design was also tested by Hampson et al. using several different configurations including the ‘racetrack’ design similar to the track system used in our study.^34^ No difference was found between the open field, where there was access to the entire field, and the racetrack system, where there was only access to the outer area, which indicates that track‐type systems may compensate for a restriction of grazing and space. Our findings, along with others such as Maisonpierre et al., support the hypothesis that intensive management and space restriction are likely to have a negative impact on the levels of voluntary exercise that domestic horses perform.^13^ Previous work has shown that, compared with an open field, strip grazing did not reduce movement and so our work implies that trackway systems actively encourage an increase in movement.^33^ Although previous evidence looking at the benefits of low‐intensity exercise in horses is conflicting, there is support that it may provide health benefits. de Laat et al. (2016) demonstrated that the use of dynamic feeding systems, designed to increase movement, over a 3‐month period resulted in a decrease in body fat and an improvement in BCS.^35^ It has also been shown that low‐intensity exercise programmes, with and without concurrent calorie restriction, resulted in improvements in metabolic health and systemic inflammatory biomarkers compared with dietary restriction alone.^36^, ^37^ We found that ambulation increased with each passing week up until Week 3 on the track system, which may be explained by habituation as the ponies become increasingly comfortable and thus explorative in their environment, which may be more limited in the strip system. This bears consideration when determining the impact of systems like rotational grazing, which may result in relocation so frequently that habituation is compromised. However, it should be noted that confounding factors, such as environmental factors which impact movement, were not accounted for in this study.

Ponies on the track system engaged in more categories of behaviours, which we considered to be most indicative of welfare status, potentially indicating a more diverse behavioural repertoire being displayed. This could be due to a more enriched environment, less competition over resources or improved social cohesion. We also found that allogooming behaviour declined over the first 3 weeks across both systems. Although generally associated with positive social interactions,^38^ allogrooming rates have been shown to correlate negatively with the proportion of adult horses in a group which may be due to a lack of requirement to improve social relations when they are already well established.^39^ The witnessed initial increases in rates of allogrooming seen in this study are, therefore, more likely to reflect a breakdown in social cohesion, given the concurrent increase in agonistic interactions, rather than an improvement.

The overall rate of overt aggression that we observed was low compared with other published data,^39^, ^40^ which may be due to our exclusion of more subtle signs of agonistic communication and limitation to overt behaviours. However, Sigurjónsdóttir and Haraldsson reported that stability of group membership is strongly correlated with lower aggression and the groups included in our study were well established, so a low rate of agonistic behaviour would be expected.^39^ We observed a higher rate of overt agonistic interactions in the strip system, which may be due to a perceived restriction of space and a spatial concentration of resources resulting in increased competition or forced close proximity, as found in other species (e.g., pigs and automated feeders, or mice and enrichment etc.),^41^, ^42^ and likely indicates increased environmental stressors as other known stressors, such as group composition, did not change.^39^ Stereotypic behaviour, crib biting, was only observed in one pony during our study. This was a surprising and notable result as the carers of the ponies reported never having witnessed it previously, and generally, the risk factors for stereotypical behaviours were very low in the population observed. It was observed in Weeks 1 and 2 on the strip system and never on the track system. This result may lead us to further examine the relationship between stereotypical behaviours and management systems.

The ponies in this study demonstrated a significant diurnal pattern to grazing, with most grazing taking place in the morning period. The grazing system did not affect this rhythm. To our knowledge this is the first description of such a rhythm in domesticated horses and reflects similar patterns seen in wild horses^43^, ^44^, ^45^ and farmed sheep and cattle.^46^, ^47^ Our observation has several implications, clearly stabling, meal feeding or not providing ad‐lib access to forage horses will disrupt this natural rhythm, as may allowing horses to graze only at night. It is clear from human work that disruption to circadian rhythms or reversal of diurnal patterns increases metabolic risk as evidenced by work on shift workers,^48^ more work is required to determine the physiological and potential psychological impact of rhythm disruption in horses.

To conclude, the findings of this study support the hypothesis that more restrictive management practices, in this case, a strip grazing system, can have a negative impact on the behaviour and welfare of ponies, and it is important to consider this when designing and implementing weight management programmes. This reflects the preliminary findings of Mitson and Greening demonstrating that track systems may promote more movement and positive welfare compared with strip systems.^23^ As well as promoting better welfare, there may be additional physical health benefits to increasing movement that we failed to demonstrate over the time period assessed in this study. Further research is therefore needed to look at the potential physical health benefits of different grazing systems, including morphometric measurements and biomarkers of metabolic function, over extended periods of time. It is worth noting that any such research would benefit from being over a significant duration of time, potentially even multiple grazing seasons, as these systems are intended for more long‐term management changes to control weight rather than reactive dieting in the face of obesity‐related ill‐health.

## AUTHOR CONTRIBUTIONS

**Roxane Kirton:** Conceptualization; investigation; writing – original draft; methodology; validation; visualization; writing – review and editing; formal analysis; data curation. **Imogen Sandford:** Writing – original draft; investigation; data curation; formal analysis. **Eleanor Raffan:** Supervision; writing – review and editing. **Sarah Hallsworth:** Investigation; resources; project administration; methodology. **Oliver H. P. Burman:** Supervision; conceptualization; writing – review and editing. **Ruth Morgan:** Writing – review and editing; writing – original draft; formal analysis; supervision.

## FUNDING INFORMATION

Not applicable.

## CONFLICT OF INTEREST STATEMENT

The authors declare no conflict of interest.

## DATA INTEGRITY STATEMENT

Roxane Kirton and Ruth Morgan had full access to all the data in the study and takes responsibility for the integrity of the data and the accuracy of data analysis.

## ETHICAL ANIMAL RESEARCH

This study was approved by a local ethics review at the University of Lincoln and the Board of Trustees at Redwing's Horse Sanctuary.

## INFORMED CONSENT

Explicit consent was obtained from the Board of Trustees at Redwing's Horse Sanctuary.

## Supporting information


**Table S1.** Definitions of behaviours observed within the study.

## Data Availability

The data that support the findings of this study are openly available in FigShare at https://doi.org/10.58073/SRUC.25540165.v1.

## References

[1] Robin CA , Ireland JL , Wylie CE , Collins SN , Verheyen KLP , Newton JR . Prevalence of and risk factors for equine obesity in Great Britain based on owner‐reported body condition scores. Equine Vet J. 2015;47(2):196–201. 24735219 10.1111/evj.12275

[2] Morgan RA , Keen JA , McGowan CM . Treatment of equine metabolic syndrome: a clinical case series. Equine Vet J. 2015;48:422–426. 25808563 10.1111/evj.12445

[3] Giles SL , Rands SA , Nicol CJ , Harris PA . Obesity prevalence and associated risk factors in outdoor living domestic horses and ponies. PeerJ. 2014;20:e299. 10.7717/peerj.299 PMC3970797 24711963

[4] Pollard D , Wylie CE , Verheyen KLP , Newton JR . Identification of modifiable factors associated with owner‐reported equine laminitis in Britain using a web‐based cohort study approach. BMC Vet Res. 2019;15:59. 30755193 10.1186/s12917-019-1798-8 PMC6373032

[5] Argo CMCG , Curtis GC , Grove‐White D , Dugdale AHA , Barfoot CF , Harris PA . Weight loss resistance: a further consideration for the nutritional management of obese Equidae. Vet J. 2012;194:179–188. 23117030 10.1016/j.tvjl.2012.09.020

[6] Hockenhull J , Creighton E . The day‐to‐day management of UK leisure horses and the prevalence of owner reported stable‐related and handling behaviour problems. Animal Welfare. 2015;24:29–36.

[7] Durham AE , Frank N , McGowan CM , Menzies‐Gow NJ , Roelfsema E , Vervuert I , et al. ECEIM consensus statement on equine metabolic syndrome. J Vet Intern Med. 2019;33:335–349. 30724412 10.1111/jvim.15423 PMC6430910

[8] Argo CM , Dugdale HA , McGowan CM . Considerations for the use of restricted, soaked grass hay diets to promote weight loss in the management of equine metabolic syndrome and obesity. Vet J. 2015;206:170–177. 26403956 10.1016/j.tvjl.2015.07.027

[9] Dugdale AH , Curtis GC , Cripps P , Harris PA , Argo CM . Effect of dietary restriction on body condition, composition and welfare of overweight and obese pony mares. Equine Vet J. 2010;42:600–610. 20840575 10.1111/j.2042-3306.2010.00110.x

[10] Gill JC , Pratt‐Phillips SE , Mansmann R , Siciliano PD . Weight loss Management in Client‐Owned Horses. J Equine Vet Sci. 2016;39:80–89.

[11] McGowan CM , Dugdale AH , Pinchbeck GL , Argo CM . Dietary restriction in combination with a nutraceutical supplement for the management of equine metabolic syndrome in horses. Vet J. 2013;196:153–159. 23141962 10.1016/j.tvjl.2012.10.007

[12] Boyd LE , Carbonaro DA , Houpt KA . The 24‐hour time budget of Przewalski horses. Appl Anim Behav Sci. 1988;21:5–17.

[13] Maisonpierre IN , Sutton MA , Harris P , Menzies‐Gow N , Weller R , Pfau T . Accelerometer activity tracking in horses and the effect of pasture management on time budget. Equine Vet J. 2019;51:840–845. 31009100 10.1111/evj.13130

[14] Jørgensen GHM , Borsheim L , Mejdell CM , Søndergaard E , Bøe KE . Grouping horses according to gender—effects on aggression, spacing and injuries. Appl Anim Behav Sci. 2009;120:94–99.

[15] Hughes BO , Duncan IJH . The notion of ethological ‘need’, models of motivation and animal welfare. Anim Behav. 1988;36:1696–1707.

[16] Hothersall B , Casey R . Undesired behaviour in horses: a review of their development, prevention, management and association with welfare. Equine Vet Educ. 2012;24:479–485.

[17] Saris WH . Very‐low‐calorie diets and sustained weight loss. Obes Res. 2001;9(S4):295s–301s. 11707557 10.1038/oby.2001.134

[18] Cameron A , Harris P , Longland A , Horseman S , Hockenhull J . UK horse carers' experiences of restricting grazing when aiming to prevent health issues in their horses. J Equine Vet Sci. 2021;104:103685. 34417001 10.1016/j.jevs.2021.103685

[19] Longland AC , Barfoot C , Harris PA . Strip grazing: changes in biomass, nutrient content and digestibility of temperate, midsummer pasture by strip‐grazed or ‘free’‐grazing ponies, over 4 weeks. J Equine Vet Sci. 2023;131:104957. 37890600 10.1016/j.jevs.2023.104957

[20] Longland AC , Barfoot C , Harris PA . Strip‐grazing: reduces pony dry matter intakes and changes in bodyweight and morphometrics. Equine Vet J. 2022;54:159–166. 33369770 10.1111/evj.13416

[21] Furtado T , King M , Perkins E , McGowan C , Chubbock S , Hannelly E , et al. An exploration of environmentally sustainable practices associated with alternative grazing management system use for horses, ponies, donkeys and mules in the UK. Animals. 2022;12(2):151. 35049774 10.3390/ani12020151 PMC8772570

[22] Hampson BA , Morton JM , Mills PC , Trotter MG , Lamb DW , Pollitt CC . Monitoring distances travelled by horses using GPS tracking collars. Aust Vet J. 2010;88:176–181. 20529024 10.1111/j.1751-0813.2010.00564.x

[23] Mitson K , Greening L . A preliminary investigation comparing the frequency of grazing and movement behavior between a track paddock system and a conventional paddock system. J Vet Behav. 2019;29:155.

[24] Carroll CL , Huntington PJ . Body condition scoring and weight estimation of horses. Equine Vet J. 1988;20:41–45. 3366105 10.1111/j.2042-3306.1988.tb01451.x

[25] Carter RA , Geor RJ , Burton Staniar W , Cubitt TA , Harris PA . Apparent adiposity assessed by standardised scoring systems and morphometric measurements in horses and ponies. Vet J. 2009;179:204–210. 18440844 10.1016/j.tvjl.2008.02.029

[26] Altmann J . Observational study of behavior: sampling methods. Behaviour. 1974;49:227–267. 4597405 10.1163/156853974x00534

[27] Boissy A , Manteuffel G , Jensen MB , Moe RO , Spruijt B , Keeling LJ , et al. Assessment of positive emotions in animals to improve their welfare. Physiol Behav. 2007;92:375–397. 17428510 10.1016/j.physbeh.2007.02.003

[28] Dalla Costa E , Murray L , Dai F , Canali E , Minero M . Equine on‐farm welfare assessment: a review of animal‐based indicators. Anim Welf. 2014;23:323–341.

[29] Hockenhull J , Whay HR . A review of approaches to assessing equine welfare. Equine Vet Educ. 2014;26:159–166.

[30] Morrison PK , Harris PA , Maltin CA , Grove‐White D , Barfoot CF , Argo CM . Perceptions of obesity and management practices in a UK population of leisure‐horse owners and managers. J Equine Vet Sci. 2017;53:19–29.

[31] Harris PA , Ellis AD , Fradinho MJ , Jansson A , Julliand V , Luthersson N , et al. Review: feeding conserved forage to horses: recent advances and recommendations. Animal. 2017;11:958–967. 27881201 10.1017/S1751731116002469

[32] Harris P . Nutrition, behaviour and the role of supplements for calming horses: the veterinarian's dilemma. Vet J. 2005;170:10–11. 15993785 10.1016/j.tvjl.2004.08.007

[33] Cameron A , Longland A , Pfau T , Pinnegar S , Brackston I , Hockenhull J , et al. The effect of strip grazing on physical activity and behavior in ponies. J Equine Vet Sci. 2022;110:103745. 34972031 10.1016/j.jevs.2021.103745

[34] Hampson BA , de Laat MA , Mills PC , Pollitt CC . Distances travelled by feral horses in ‘outback’ Australia. Equine Vet J Suppl. 2010;42(S38):582–586. 10.1111/j.2042-3306.2010.00203.x 21059064

[35] de Laat MA , Hampson BA , Sillence MN , Pollitt CC . Sustained, low‐intensity exercise achieved by a dynamic feeding system decreases body fat in ponies. J Vet Intern Med. 2016;30:1732–1738. 27639952 10.1111/jvim.14577 PMC5032883

[36] Bamford NJ , Potter SJ , Baskerville CL , Harris PA , Bailey SR . Influence of dietary restriction and low‐intensity exercise on weight loss and insulin sensitivity in obese equids. J Vet Intern Med. 2019;33:280–286. 30520164 10.1111/jvim.15374 PMC6335535

[37] Moore JL , Siciliano PD , Pratt‐Phillips SE . Effects of diet versus exercise on morphometric measurements, blood hormone concentrations, and oral sugar test response in obese horses. J Equine Vet Sci. 2019;78:38–45. 31203982 10.1016/j.jevs.2019.03.214

[38] Hall C , Randle H , Pearson G , Preshaw L , Waran N . Assessing equine emotional state. Appl Anim Behav Sci. 2018;205:183–193.

[39] Sigurjónsdóttir H , Haraldsson H . Significance of group composition for the welfare of pastured horses. Animals. 2019;9(1):14. 30621272 10.3390/ani9010014 PMC6356279

[40] Fureix C , Bourjade M , Henry S , Sankey C , Hausberger M . Exploring aggression regulation in managed groups of horses Equus caballus. Appl Anim Behav Sci. 2012;138:216–228.

[41] Georgsson L , Svendsen J . Degree of competition at feeding differentially affects behavior and performance of group‐housed growing‐finishing pigs of different relative weights. J Anim Sci. 2002;80:376–383. 11881927 10.2527/2002.802376x

[42] Nip E , Adcock A , Nazal B , MacLellan A , Niel L , Choleris E , et al. Why are enriched mice nice? Investigating how environmental enrichment reduces agonism in female C57BL/6, DBA/2, and BALB/c mice. Appl Anim Behav Sci. 2019;217:73–82.

[43] Berger A , Scheibe K‐M , Eichhorn K , Scheibe A , Streich J . Diurnal and ultradian rhythms of behaviour in a mare group of Przewalski horse (*Equus ferus przewalskii*), measured through one year under semi‐reserve conditions. Appl Anim Behav Sci. 1999;64:1–17.

[44] Mayes E , Duncan P . Temporal patterns of feeding behaviour in free‐ranging horses. Behaviour. 1986;96:105–129.

[45] Ralston SL , Van den Broek G , Baile CA . Feed intake patterns and associated blood glucose, free fatty acid and insulin changes in ponies. J Anim Sci. 1979;49:838–845. 393688 10.2527/jas1979.493838x

[46] Sheahan AJ , Boston RC , Roche JR . Diurnal patterns of grazing behavior and humoral factors in supplemented dairy cows. J Dairy Sci. 2013;96:3201–3210. 23453522 10.3168/jds.2012-6201

[47] Plaza J , Palacios C , Abecia JA , Nieto J , Sánchez‐García M , Sánchez N . GPS monitoring reveals circadian rhythmicity in free‐grazing sheep. Appl Anim Behav Sci. 2022;251:105643.

[48] Mohd Azmi NAS , Juliana N , Mohd Fahmi Teng NI , Azmani S , Das S , Effendy N . Consequences of circadian disruption in shift workers on chrononutrition and their psychosocial well‐being. Int J Environ Res Public Health. 2020;17:E2043. 10.3390/ijerph17062043 PMC7142532 32204445

