# Supplementary material for: The impact of restricted grazing systems on the behaviour and welfare of ponies
Source: Equine Vet J. 2024 Sep 14;57(3):737–44. doi: 10.1111/evj.14411 (PMC11982426; doi:10.1111/evj.14411)
Supplement: Supplementary file 1 — Table S1. Definitions of behaviours observed within the study. [file EVJ-57-737-s001.pdf]

Table S1: Definitions of behaviours observed within the study.

**Behaviour**	**Definition**
Grazing	Eating grass and other low-lying vegetation from the ground
Browsing	Eating higher growing plant materials such as hedging and trees
Walking	Moving in a forward direction with a four-beat gait
Trotting	Moving in a forward direction with a two-beat gait
Cantering	Moving in a forward direction with a three-beat gait
Galloping	Moving in a forward direction with a rapid four-beat gait
Ambulating	Moving in either walk, trot, canter or gallop
Drinking	Consuming water
Standing	Standing without moving
Sternal recumbency	Lying with the sternum and ventral abdomen in contact with the ground
Lateral recumbency	Lying on the side with the body wall, neck and head in contact with the ground
Self-grooming	Using the teeth or hooves to scratch an area of the body or rubbing against an object
Urination	Passing urine
Defecation	Passing faeces
Play	Engaging in object, locomotory, play fighting or sexual play behaviours
Allogrooming	Reciprocated grooming of another individual
Overt agonistic interactions	Any clearly observable agonistic interactions including biting, bite threats, lunging, striking, kicking and kick threats
Stereotypic behaviours	Any abnormal repetitive behaviours lacking a clear function, including locomotory and oral behaviours
